# Efficacy of Cyanoacrylate Tissue Adhesive for Closure of Laparoscopic Trocar Sites: A Single-Center, Prospective, Cohort Study

**DOI:** 10.7759/cureus.101155

**Published:** 2026-01-09

**Authors:** Mukteshwar Deshmukh, Srinath Amudan, Viplava Thakur, Siddesh Sonawane

**Affiliations:** 1 General Surgery, Indira Gandhi Government Medical College and Hospital, Nagpur, IND

**Keywords:** cyanoacrylate, laparoscopic wound closure, skin adhesive, tissue adhesive, trocar-site incision

## Abstract

Background

The application of tissue adhesives in the closure of laparoscopic trocar sites has been extensively studied in Western populations. However, evidence from India is scarce despite significant differences in patient demographics, surgical practices, and resource availability.

Methods

A prospective, single-center, cohort study was conducted at a tertiary care center from November 2022 to July 2025. Patients eligible to undergo an elective laparoscopic procedure and trocar site closure with n-butyl-cyanoacrylate tissue adhesive were included in this study. The primary co-endpoints of the study were wound closure time, cost-effectiveness, and wound dehiscence.

Results

A total of 114 patients were included in this analysis, of which 68 (59.64%) were male. The majority of the patients, i.e., 32 (28.07%), belonged to the 21-30 years age group. The most common type of laparoscopic surgery was appendectomy, performed in 60 (52.63%) patients. The port size was 5 mm in 165 (45%) patients and 10 mm in 202 (55%) patients. The mean closure time increased with the number of ports, from 20 seconds in single-port closures to 90 seconds in five-port closure surgeries. Complete hemostasis was achieved in 98 (85.95%) patients. Postoperative complications such as wound dehiscence and discharge were observed in three (2.63%) patients. The tissue adhesive cost was 300 INR (approximately 3.5 USD) for 15 ml.

Conclusion

Surgical adhesives have been emerging as a viable alternative to suturing in small incisions, such as laparoscopic trocar sites.

## Introduction

Trocar sites are small incisions made in the skin and underlying tissue to enable the insertion of surgical instruments during minimally invasive procedures, such as laparoscopy. These ports provide access for visualization, dissection, and repair inside the body with minimal disruption to surrounding tissues. Depending on the procedure, multiple trocar sites may be used, often ranging from 5 to 12 mm in size. Given their small size and frequent use, effective closure of trocar sites is important to minimize complications such as herniation, infection, and poor cosmetic results [1].

Cyanoacrylate tissue adhesives are liquid monomers that polymerize on contact with fluid or basic medium and form a strong bond when applied to moist skin. The earlier short-chain tissue adhesives, i.e., methyl-2 and ethyl-2-cyanoacrylate, although effective, had limited clinical application due to rapid degradation, causing significant toxicity leading to acute and chronic inflammation. In contrast, longer-chain derivatives such as n-butyl-2-cyanoacrylate and 2-octyl-cyanoacrylate degrade more slowly, thus limiting the accumulation of toxic byproducts in tissues, making them safer for skin closure [2]. Tissue adhesives, therefore, offer several advantages such as rapid application, avoidance of needlestick injury, less technical demand, immediate sealing, elimination of suture removal, comparable cosmetic outcomes, low complication rates, and limited aftercare [3,4]. Although these tissue adhesives have been extensively studied in Western populations, evidence from India is scarce, even though patient demographics, surgical practices, and resource availability differ significantly. This study was therefore undertaken to prospectively evaluate the efficacy and safety of n-butyl cyanoacrylate tissue adhesive for laparoscopic trocar site closure in the Indian population.

## Materials and methods

Study design

A prospective, single-center, single-arm cohort study was conducted at a tertiary care center from November 2022 to July 2025. The study included patients eligible to undergo elective laparoscopic procedures and trocar site closure with n-butyl-cyanoacrylate tissue adhesive. The study inclusion and exclusion criteria are presented in Table 1. The study was conducted in accordance with the principles outlined in the Declaration of Helsinki [5]. All patients were counseled about the surgical procedure, including details of the study intervention. All patients provided written informed consent to undergo the procedure and data collection, and its analysis for research purposes.

**Table 1:** Study inclusion and exclusion criteria

Inclusion criteria	Exclusion criteria
Patients from all age groups and ASA grades who are eligible to undergo elective laparoscopic procedures and trocar site closure with n-butyl-cyanoacrylate tissue adhesive	Patients with a need for emergency conversion to open surgery due to complications; with trocar sites that failed to achieve hemostasis; with a history of abdominal surgery at the proposed trocar sites; with hypersensitivity to cyanoacrylate adhesives; with contraindications to general anesthesia; with coagulopathy; with ascites; with an immunocompromised and obstetric status

Preoperative investigations

All eligible study patients scheduled for elective laparoscopic surgery underwent a comprehensive clinical evaluation. Physical examinations were also performed. Routine laboratory investigations comprising complete blood count (CBC), kidney function test (KFT), liver function test (LFT), coagulation profile, i.e., prothrombin time (PT), activated partial thromboplastin time (aPTT), and international normalized ratio were performed. Serological screening for human immunodeficiency virus (HIV) and hepatitis B surface antigen (HBsAg) was done. Twelve-lead electrocardiography, chest radiography, and ultrasonography of the abdomen and pelvis were also performed.

Study procedure

**Figure 1:**
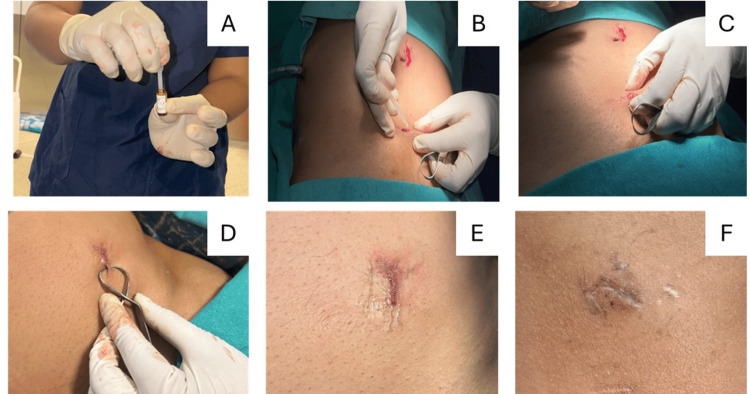
Representative images of the operative procedures A: Aspiration of cyanoacrylate glue B: Application of glue to the port site C, D: Keeping the edges approximated for 15 seconds E: Approximated edges after glue sets F: Postoperative day 7 of the port site

The surgical site was shaved before the procedure. A prophylactic intravenous dose of 2 g ceftriaxone or 2 g cefotaxime was administered at the time of induction of general anesthesia. Laparoscopic surgery was performed as per standard surgical protocols. Upon completion of the procedure, all trocar sites were assessed for bleeding. After hemostasis, trocar port wound closure was performed using n-butyl-cyanoacrylate, a synthetic topical tissue adhesive. According to the standardized technique [6] for skin adhesive application, after the procedure was performed, the wound was cleaned, and the edges of the skin were dried. The ampoule containing n-butyl-cyanoacrylate was broken, and its contents were aspirated using a sterile dropper. The skin edges were approximated and aligned using tissue forceps. The adhesive was applied along intervals where the skin edges met, and the edges were held together for 15-20 seconds to allow the adhesive to stiffen. The edges were then assessed for dehiscence. A sterile dressing with a 5x5 cm single gauze folded on itself according to the wound size was applied. Trocar sites with mild hemorrhagic ooze necessitated manual compression for two minutes, after which the site was reassessed, and tissue adhesive was reapplied (Figure 1). Specific trocar sites wherein hemostasis was not achieved even on manual compression were abandoned and sutured instead. After the procedure, all patients were monitored for port site complications.

Study endpoints

The primary endpoints of the study were wound closure time, cost-effectiveness, and wound dehiscence. Wound dehiscence was defined as a partial or complete separation of the wound edges.

Data collection

Patient demographics (age, gender, and medical history, etc.) were recorded. Intraoperative details such as type of procedure, number of ports, port size, hemostasis, and outcomes related to adhesive application were also recorded. Data collection was performed during the intraoperative period and at three days, one week, and six weeks postoperatively.

Statistical analysis

Data was analyzed using Microsoft Excel 2021/365 (Microsoft Corp., Redmond, USA). Categorical variables are presented as counts and percentages. 

## Results

Demographics of study patients

A total of 114 patients were included in the study, of which 68 (59.64%) were males. The unequal distribution of females and males in the patient group did not allow for comparative analysis between the genders. The majority of the patients (n=32, 28.07%) belonged to the 21-30 years age group. There was only one patient (0.88%) who belonged to the 0-10 years age group. The demographic details of the study patients are detailed in Table 2.

**Table 2:** Demographic details of study participants

Variable	Study patients (n=114)
Males	68 (59.64%)
Age distribution, years	
0-10	1 (0.88%)
11-20	22 (19.30%)
21-30	32 (28.07%)
31-40	21 (18.42%)
41-50	13 (11.40%)
51-60	14 (12.28%)
61-70	8 (7.02%)
71-80	3 (2.63%)

Procedural details

The most common type of laparoscopic surgery was appendectomy, performed in 60 (52.63%) patients, followed by cholecystectomy in 33 (28.95%) patients, totally extraperitoneal hernia repair in 16 (14.03%) patients, fundoplication in one (0.88%) patient, and transabdominal preperitoneal repair in one (0.88%) patient. Diagnostic laparoscopy was performed in three (2.63%) patients. A total of 367 ports were involved. Among these, 177 cases (48.43%) were related to appendectomy, 129 cases (35.15%) were associated with cholecystectomy, 48 cases (13.08%) were for totally extraperitoneal procedures, five cases (1.36%) were for fundoplication, three cases (0.82%) involved diagnostic laparoscopy with adhesiolysis, another three cases (0.82%) were for transabdominal preperitoneal procedures, and two cases (0.54%) were for diagnostic laparoscopy. The port size was 5 mm in 165 (45.00%) patients and 10 mm in 202 (55.00%) patients. The procedural details are elaborated in Table 3.

**Table 3:** Study procedural details

Variable	Number of patients (n=114)
Type of procedure	
Appendectomy	60 (52.63%)
Cholecystectomy	33 (28.95%)
Totally extra-peritoneal	16 (14.03%)
Diagnostic laparoscopy	3 (2.63%)
Fundoplication	1 (0.88%)
Transabdominal preperitoneal	1 (0.88%)
Number of ports (n=367)	
Appendectomy	177 (48.43%)
Cholecystectomy	129 (35.15%)
Totally extra-peritoneal	48 (13.08%)
Fundoplication	5 (1.36%)
Diagnostic laparoscopy with adhesiolysis	3 (0.82%)
Transabdominal preperitoneal	3 (0.82%)
Diagnostic laparoscopy	2 (0.54%)
Port size	
5 mm	165 (45.00%)
10 mm	202 (55.00%)

Study outcomes

Complete hemostasis was achieved in 98 (85.95%) patients. Mild ooze was observed in 10 (8.77%) patients. Postoperative complications such as wound gape and discharge were observed in three (2.63%) patients. The study outcomes are depicted in Table 4. The tissue adhesive used for this study cost 300 INR (approximately 3.5 USD, conversion as per current exchange rate) for 15 ml. However, the precise data on operating room costs were unavailable. Table 5 provides insight into the wound complication management on postoperative day 7 in three patients. Time of closure for laparoscopic procedures per patient (e.g., 4 ports, 3 ports, 5 ports) was recorded for all patients. However, the time of closure for the individual ports was not recorded. The average time of closure for laparoscopic procedures is depicted in Table 6.

**Table 4:** Study outcomes

Variable	Study patients (n=114)
Postoperative complications	
Wound gape	3 (2.63%)
Discharge	3 (2.63%)
Hemostasis	
Complete	98 (85.95%)
Mild ooze	10 (8.77%)
Not achieved	6 (5.26%)

**Table 5:** Postoperative day 7 complication management

Surgery	Gape	Infection	Southampton grading	Management
Patient 1, port: right iliac fossa (10 mm)				
Laparoscopic appendectomy	Yes	No	2A	Wound dressing
Patient 2, port: umbilical (10 mm)				
Laparoscopic cholecystectomy	Yes	Yes	4A	Oral antibiotics, daily dressing for three days
Laparoscopic cholecystectomy	No	Yes	4A	Oral antibiotics, daily dressing for three days
Patient 3, port: Palmer's point (10 mm)				
Diagnostic laparoscopy with adhesiolysis	Yes	Yes	4A	Oral antibiotics, daily dressing for three days

**Table 6:** Average closure time of laparoscopic procedures TAPP - transabdominal preperitoneal; TEP - totally extraperitoneal

Surgery	Average closure time (in seconds)
5 ports (laparoscopic cholecystectomy)	74.33
3 ports (laparoscopic appendectomy, laparoscopic TEP, laparoscopic TAPP, diagnostic laparoscopy with adhesiolysis)	53.93
Diagnostic laparoscopy (1 port)	20
Laparoscopic fundoplication (5 ports)	90

## Discussion

Proper wound closure is a critical aspect of wound care. The ideal wound closure method should have easy and rapid application, less pain, and minimal chances of infection, dehiscence, and scarring [7,8]. The current study prospectively evaluated the efficacy and safety of n-butyl-cyanoacrylate tissue adhesive for laparoscopic trocar site closure in the Indian population.

Wound closure time was evaluated as a primary endpoint. Sebesta et al. [3] reported significantly shorter closure time of 3 minutes 47 seconds with octyl cyanoacrylate compared to 14 minutes 5 seconds for sutures. Matin et al. [9] demonstrated mean closure times of 2 minutes 30 seconds with octyl cyanoacrylate and 6 minutes with sutures (p<0.001). Similarly, Dowson et al. [10] observed mean closure times of 125 sec with n-butyl-cyanoacrylate compared to 220 sec with sutures. Maartense et al. [11] documented wound closure times of 26 seconds for adhesive paper tape, 33 seconds for octyl cyanoacrylate, and 65 seconds for poliglecaprone, with octyl cyanoacrylate requiring the fewest procedural steps. In contrast, Deshpande et al. [12] reported no statistically significant difference between adhesives (198.4±62.96 seconds) and sutures (171.1±29.03 seconds) closure times. Pattanshetti et al. [13] also found wound closure times to be comparable between octyl cyanoacrylate and sutures.

Cost-effectiveness was a primary endpoint of the current study. A study conducted in the United States published in 2003 [3] evaluated the economic impact of using octyl cyanoacrylate compared to traditional sutures for skin incision closure. The mean cost per patient was $65.10 for octyl cyanoacrylate and $7.74 for traditional sutures. However, when factoring in time-related costs, closure with octyl cyanoacrylate averaged $128.90, compared to $490.93 for traditional suturing. This resulted in a total closure cost of $193.32 for octyl cyanoacrylate compared to $497 for traditional sutures - a statistically significant finding. Although the adhesive itself was more expensive, the significantly reduced closure time made octyl cyanoacrylate the more cost-effective option overall. A study from the Netherlands, published in 2003 [11], reported per-patient costs of €8.68 for adhesive papertape closure, €17.82 for poliglecaprone, and €34 for octyl cyanoacrylate. Octyl cyanoacrylate was significantly more expensive than both poliglecaprone and adhesive paper tape (p<0.001). A similar study from the United Kingdom, also published in 2006 [10], reported the per-unit cost of n-butyl-cyanoacrylate tissue adhesive as $8 compared to $5 for two lengths of suture material. An Indian study published in 2016 [12] found the cost of one unit of octyl cyanoacrylate tissue adhesive to be 911 INR, while a packet of non-absorbable polyamide suture cost 113 INR. Thus, the octyl cyanoacrylate tissue adhesive cost was eightfold greater than that of the suture. In the current study, Ethilon™ 2-0 suture costs 250 INR, Ethilon™ 3-0 suture costs 247 INR, and n-butyl-cyanoacrylate (0.15 ml) costs 300 INR. Although costs were comparable, wound closure with n-butyl cyanoacrylate tissue adhesive required a single dressing, which was removed on postoperative day 7, requiring no intermediate follow-up visits until the six-week follow-up. These factors contribute to the overall cost-effectiveness of tissue adhesives. Other studies have supported the findings of the aforementioned studies [14].

Wound dehiscence was the third primary endpoint. Several previous investigations have reported varying incidence rates for wound-related complications; wound dehiscence is one such complication. In a study involving 100 patients, Rosin et al. [15] reported 2% partial wound dehiscence, attributing it to poor wound edge approximation. Sebesta et al. [3] in their study comprising 59 patients reported 6.67% wound dehiscence in octyl cyanoacrylate patients. Matin et al. [9] conducted a randomized controlled trial comprising 92 patients and reported 2.0% wound dehiscence in octyl cyanoacrylate patients and 8.1% wound dehiscence in the traditional suture patients. Dowson et al. [10] in their study of 154 patients documented 5.26% wound dehiscence 24-48 hours postoperatively in n-butyl-cyanoacrylate patients. There were no incidences of wound dehiscence in the traditional suture patients. However, during the 4-6-week postoperative time, 6.56% and 3.45% occurrences of wound dehiscence in the n-butyl-cyanoacrylate tissue adhesive and traditional suture patients were observed, respectively. Deshpande et al. [12], in a small cohort of 20 patients, observed 40% wound dehiscence in octyl cyanoacrylate patients and 20% wound dehiscence in suture patients on postoperative day 7. Similarly, Deolekar et al. [16] reported 8% wound dehiscence in octyl cyanoacrylate patients. Qureshi et al. [17] observed 0.8% wound dehiscence in transverse abdominal wounds >10 cm in n-butyl-cyanoacrylate tissue adhesive.

Cosmetic outcomes could not be evaluated in this study, and we acknowledge this as a limitation. Nonetheless, multiple prior studies have reported that tissue adhesives generally yield superior cosmetic results compared with traditional sutures and staples [18-20]. However, there are a few studies that have found these methods to be at par with each other [21-25].

The current study has a few limitations. An average wound closure time for each type of laparoscopic surgery was recorded; however, the closure time for each port site could not be measured. In our setting, where non-absorbable simple interrupted sutures are routinely used and precise data on operating room costs are unavailable, total cost measurements were not feasible. We did not have a cosmetic outcome as the primary endpoint for this study. Nonetheless, multiple prior studies have reported that tissue adhesives generally yield comparable or superior cosmetic results to traditional sutures and staples. This was a preliminary single-center study, without prior sample size calculation or power analysis. Therefore, future large-scale prospective randomized controlled trials in an Indian setting are warranted to provide further insights.

## Conclusions

Surgical adhesives have been emerging in recent times as a good alternative to suturing in small incisions like laparoscopic trocar sites. They are reported to have comparable wound healing and infection rates when adhesives are used appropriately. They are efficient, provide shorter closure times, and are cost-effective. Hence, it is a viable alternative for closing laparoscopic wounds. Moreover, its ease of application reduces the need for specialized suturing skills, making it particularly useful in high-volume surgical settings.
